# Age constraint for the Moreno Hill Formation (Zuni Basin) by CA-TIMS and LA-ICP-MS detrital zircon geochronology

**DOI:** 10.7717/peerj.10948

**Published:** 2021-03-09

**Authors:** Charl D. Cilliers, Ryan T. Tucker, James L. Crowley, Lindsay E. Zanno

**Affiliations:** 1 Department of Earth Sciences, Faculty of Science, Stellenbosch University, Stellenbosch, Western Cape, South Africa; 2 Department of Geosciences, Boise State University, Boise, ID, USA; 3 Paleontology, North Carolina Museum of Natural Sciences, Raleigh, NC, USA; 4 Department of Biological Sciences, North Carolina State University, Raleigh, NC, USA

**Keywords:** Moreno Hill Formation, Zuni Basin, Mogollon Highlands, Coupled CA-TIMS and LA-ICP-MS, Gallup Delta, Detrital zircon

## Abstract

The “mid-Cretaceous” (~125–80 Ma) was punctuated by major plate-tectonic upheavals resulting in widespread volcanism, mountain-building, eustatic sea-level changes, and climatic shifts that together had a profound impact on terrestrial biotic assemblages. Paleontological evidence suggests terrestrial ecosystems underwent a major restructuring during this interval, yet the pace and pattern are poorly constrained. Current impediments to piecing together the geologic and biological history of the “mid-Cretaceous” include a relative paucity of terrestrial outcrop stemming from this time interval, coupled with a historical understudy of fragmentary strata. In the Western Interior of North America, sedimentary strata of the Turonian–Santonian stages are emerging as key sources of data for refining the timing of ecosystem transformation during the transition from the late-Early to early-Late Cretaceous. In particular, the Moreno Hill Formation (Zuni Basin, New Mexico) is especially important for detailing the timing of the rise of iconic Late Cretaceous vertebrate faunas. This study presents the first systematic geochronological framework for key strata within the Moreno Hill Formation. Based on the double-dating of (U-Pb) detrital zircons, via CA-TIMS and LA-ICP-MS, we interpret two distinct depositional phases of the Moreno Hill Formation (initial deposition after 90.9 Ma (middle Turonian) and subsequent deposition after 88.6 Ma (early Coniacian)), younger than previously postulated based on correlations with marine biostratigraphy. Sediment and the co-occurring youthful subset of zircons are sourced from the southwestern Cordilleran Arc and Mogollon Highlands, which fed into the landward portion of the Gallup Delta (the Moreno Hill Formation) via northeasterly flowing channel complexes. This work greatly strengthens linkages to other early Late Cretaceous strata across the Western Interior.

## Introduction

**Figure 1:**
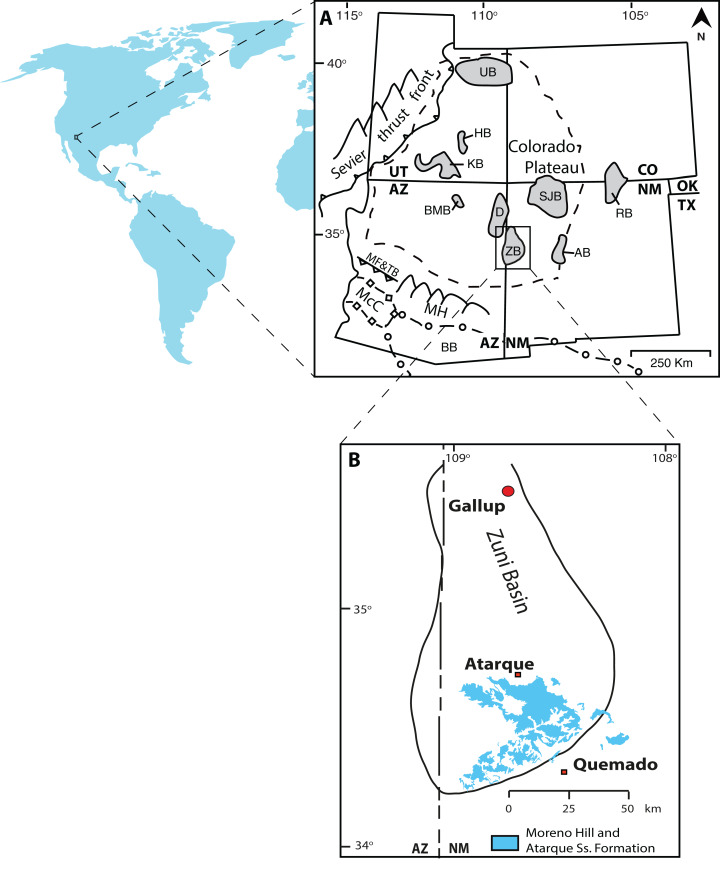
Location map. (A) Map of Colorado Plateau and adjacent areas (modified from Molenaar (1983a), Dickinson et al. (2012), Pecha et al. (2018)); (B) Map of the Zuni Basin and exposure of the Atarque Sandstone and Moreno Hill Formation (modified after Trumbull (1960), McLellan et al. (1983b), New Mexico Bureau of Geology & Mineral Resources (2003), and references therein). Note: UB, Uinta Basin; HB, Henry Basin; KB, Kaiparowits Basin; RB, Raton Basin; SJB, San Juan Basin; BMB, Black Mesa Basin; ZB, Zuni Basin; AB, Acoma Basin; McC, McCoy Basin; BB, Bisbee Basin; D, Defiance Uplift; MH, Mogollon Highlands; MF&TB, Maria Fold and Thrust Belt.

The Moreno Hill Formation (Zuni Basin, New Mexico) provides an important snapshot of North America’s late-Early to early-Late Cretaceous Mesozoic sediment history. The Moreno Hill Formation and the larger Zuni Basin (Fig. 1) document a dynamic period of tectonic upheaval, active volcanism, greenhouse climate, and an ever-changing coastal margin in North America (Kauffman & Hart, 1996; Lloyd et al., 2008; Benton, 2009; Chaboureau et al., 2014; Haq, 2014; Huber et al., 2018). Specifically, the western margin of the Western Interior Seaway underwent significant shifts in coastal morphology and coupled with on-going tectonism resulted in numerous unconformities, lateral discontinuities, and cryptic or tenuous stratigraphic linkages (Molenaar, 1983a; Molenaar et al., 2002; Haq, 2014; Albright & Titus, 2016; D’Emic et al., 2019). Although a number of studies have sought to resolve these complexities by obtaining depositional ages via radiometric age dating, the “mid-Cretaceous” southwestern shoreline (New Mexico) of the Western Interior Seaway has largely gone underexplored (Lawton & Bradford, 2011; Meyers et al., 2012; Roberts et al., 2013; Barclay et al., 2015; Juárez-Arriaga et al., 2019; Lawton, 2019; Laurin et al., 2019; Pană, Poulton & DuFrane, 2019; Rinke-Hardekopf, Dashtgard & MacEachern, 2019). Adjacent basins offer little aid as lithostratigraphic relationships become increasingly cryptic (Albright & Titus, 2016; D’Emic et al., 2019; Tucker et al., 2020).

Furthermore, recent studies have sought to address the variable relationship of volcaniclastic (primary-deposit) and volcanilithic (secondary-deposit) rock used to date sedimentary sequences (Rossignol et al., 2019; Tucker et al., 2020). This is increasingly important, particularly when detangling the complexities of Maximum Depositional Ages (MDAs) and ecosystem evolution within terrestrial depo-centers that are marine-adjacent, including possible linkages to the Gallup Delta (Jinnah et al., 2009; Chure et al., 2010; Irmis et al., 2011; Ross et al., 2017; Coutts, Matthews & Hubbard, 2019; Herriott et al., 2019; Lin & Bhattacharya, 2019; Lin, Bhattacharya & Stockford, 2019; Joeckel et al., 2020; Tucker et al., 2020).

Based on established linkages to marine biostratigraphic records tied to radiometric dates derived from co-occurring bentonites, previously reported ages for the terrestrial Moreno Hill Formation place its deposition entirely within the Turonian (McLellan et al., 1983a; Wolfe & Kirkland, 1998; Molenaar et al., 2002; McDonald, Wolfe & Kirkland, 2006, 2010; Fowler, 2017); yet, based on stratigraphic position (overlying the Atarque Sandstone) the lowerMoreno Hill Formation was temporally assigned to the middle to upper Turonian (Pike, 1947; McLellan et al., 1983a; Wolfe & Kirkland, 1998; McDonald, Wolfe & Kirkland, 2006, Fig. 1, p. 278; McDonald, Wolfe & Kirkland, 2010). More recently, Chin et al. (2019) noted the possibility that the uppermost Moreno Hill may be younger than Turonian.

Temporal placement of the Atarque Sandstone is linked to the *Collignoniceras woollgari* biozone coupled with the occurrence of the middle Turonian ammonite *Collignoniceras woollgari woollgari* and a *Mytiloides* bivalve (reported as either *M. labiatus* or *M. mytiloides*) (Cobban & Hook, 1983; Wolfe & Kirkland, 1998; Kirkland, Smith & Wolfe, 2005). The emplacement of sediment into the Atarque archive is documented to have occurred during a “local” regressional phase linked to the tectonic influence of the ancestral Mogollon Highlands, predating the regional Western Interior Seaway regression (Wolfe, 1989; Elder & Kirkland, 1993; Molenaar, 1983a; Elder & Kirkland, 1994; Wolfe & Kirkland, 1998; Hook & Cobban, 2013). In establishing age constraints for the Moreno Hill Formation, Wolfe & Kirkland (1998, Fig. 3, p. 306, modified from Obradovich, 1993) rely on ^40^Ar/^39^Ar dates linked to ammonite biostratigraphic zones. Re-calibrations of these dates (from the Ferron Sandstone, the lower Juana Lopez beds of the D-Cross Tongue of the Mancos Shale, and from the Marias River Shale) place the Moreno Hill Formation between 91.1 ± 0.5 Ma (*Prionocyclus hyatti*) and 88.9 ± 0.6 Ma (*Scaphites preventricosus*) (Fowler, 2017). In spite of this biostratigraphic framework, correlation between terrestrial and marine sequences is challenging. Whilst the Western Interior Seaway has produced one of the most robust marine biostratigraphic frameworks in the world (Obradovich, 1993; Cobban et al., 2006), pervasive inconsistencies between key terrestrial sediment archives remain a major obstacle (Cifelli et al., 1997, 1999; Albright & Titus, 2016; D’Emic et al., 2019).

Confident age constraints for the Moreno Hill Formation are key to detailing patterns of vertebrate evolution and biostratigraphy in the early-Late Cretaceous given its well-preserved floral (angiosperm, gymnosperm) and faunal (osteichthyan fish, squamate, trionychid, crocodilian, dinosaurian and mammalian) assemblages (Wolfe et al., 1997, 2007; Wolfe & Kirkland, 1998; Kirkland & Wolfe, 2001; McDonald, Wolfe & Kirkland, 2006, 2010; Sweeney et al., 2009; Wolfe & Wolfe, 2016; Chin et al., 2019; Nesbitt et al., 2019). Whilst previously identified ash beds (tonstein or paratonstein (Admakin, 2001)) occur within its coal seams (Hoffman, 1996), these are stratigraphically limited to the lower portions of the formation. To provide broader context, we focused on utilizing U-Pb detrital geochronology on suspected near-syndepositional igneous (well-faceted) zircon grains from channel sandstone deposits in the key members spanning the Moreno Hill Formation. The well-documented detrital zircon record of the Western Interior is a crucial foundation to assess likely source terranes for these grains that are likely derived from various volcanic inliers of the westerly-adjacent Cordilleran Volcanic Arc (Willis, 1999; Dickinson & Gehrels, 2003, 2009a, 2009b; DeCelles, 2004; Laskowski, DeCelles & Gehrels, 2013). By assessing the zircon age spectra via Laser Ablation-Inductively Coupled Plasma-Mass Spectrometry (LA-ICP-MS) and subsequently dating the youngest grains via Chemical Abrasion Isotope Dilution Thermal Ionization Mass Spectrometry (CA-TIMS), this study provides the needed MDAs that the Moreno Hill Formation is currently lacking. With these MDAs we are be able to (1) refine the depositional age and duration of sedimentation of the Moreno Hill Formation; (2) provide newly calibrated linkages based on the revised temporal framework; and (3) determine likely source terranes and provide a reliable reconstruction for its emplacement. This refined temporal context will provide crucial context for paleobiogeographic and macroevolutionary studies involving terrestrial vertebrate assemblages during the mid-Cretaceous.

## Background

Subduction of the Farallon Plate under North America during the Middle Jurassic to Early Cenozoic, resulted in the Cordilleran Magmatic Arc (Miall et al., 2008; DeCelles & Graham, 2015). This, in turn, resulted in thin-skinned thrusts of the Sevier Orogeny and the Western Interior Basin, which was subsequently flooded by a vast epicontinental seaway for much of the Cretaceous (Kauffman & Caldwell, 1993; Miall et al., 2008; DeCelles & Graham, 2015). Subduction angles shallowed during the Late Campanian (Laramide Orogeny) resulting in partitioning of the Western Interior Basin into a mosaic of sub-basins preserving variable paleoenvironmental archives (Kauffman & Caldwell, 1993; Miall et al., 2008; Titus, Roberts & Albright, 2013; Lawton, 2019). Alluvial sediments within these basins contain siliciclastic, volcanic, and plutonic detritus from the westerly Cordilleran and Sevier orogenic processes and from the Mogollon Highlands to the south, a topographic feature uplifted during several Mesozoic tectonic events (Cumella, 1983; Molenaar, 1983a; Kauffman, 1984; Bilodeau, 1986; Dickinson & Gehrels, 2008; Salem, 2009; Miall & Catuneanu, 2019).

Transgressive-regressive cycles of the Western Interior Seaway, including the Greenhorn and Niobrara cyclothems, then greatly affected the extent and character of coastal habitats (Kauffman, 1969, 1977, 1984; Molenaar, 1983a; McDonough & Cross, 1991; Kauffman & Caldwell, 1993; Roberts & Kirschbaum, 1995; Miall & Catuneanu, 2019).

**Figure 2:**
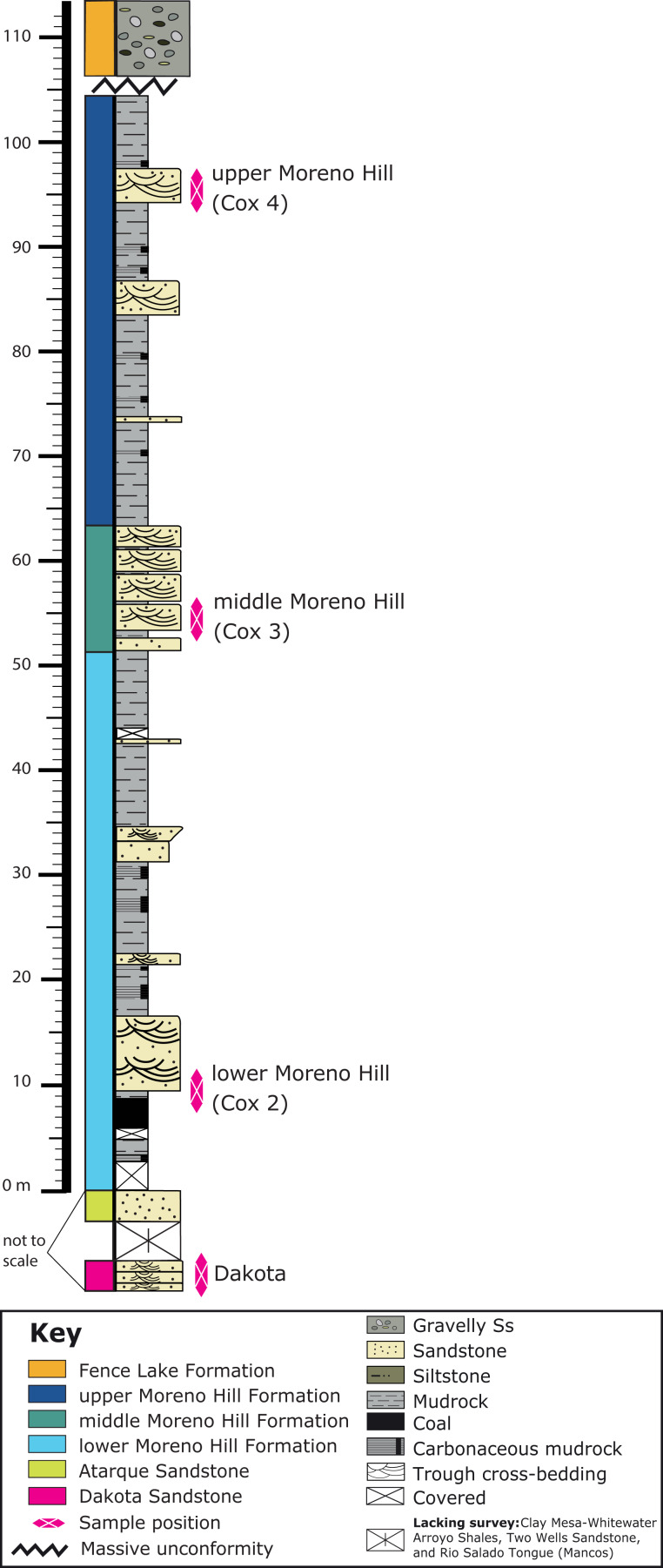
Stratigraphic column of the Moreno Hill Formation. The Moreno Hill Formation with the underlying Atarque Sandstone, underlying unsurveyed marine units and basal Cretaceous Dakota Sandstone along with the unconformably overlying Fence Lake Formation. Key stratigraphic levels sampled for detrital zircon include: (1) Dakota Sandstone; (2) lower Moreno Hill (Cox 2); (3) middle Moreno Hill (Cox 3); and (4) upper Moreno Hill (Cox 4). Whilst this stratigraphic column is based on our own measurements and observations the original type section and principle reference section stratigraphic columns for the Moreno Hill Formation were designated by McLellan et al. (1983a).

The Moreno Hill Formation is preserved in the Laramidian Zuni Basin, located between the Nutria Monocline, Zuni and Defiance uplifts, Chaco and Mogollon slopes (Kelley, 1957, 1967; McLellan et al., 1983a; Chamberlin & Anderson, 1989; Craigg, 2001; Molenaar et al., 2002). This terrestrial unit is typically exposed at surface near to Atarque (abandoned), Fence Lake, and Quemado, New Mexico, with thickness ranging from ~4.75–261.5 m (Campbell, 1981, 1984, 1987; Anderson, 1982a, 1982b, 1983; Campbell & Roybal, 1984; Hoffman, 1994). The Moreno Hill Formation, characterized by thickly bedded muds and associated channel sandstones, was originally named for deposits on the slopes of Zuni Plateau and Santa Rita Mesa (north of Zuni Salt Lake) (McLellan et al., 1983a and references therein). In section, the lower Moreno Hill overlies the shoreface deposits of the Atarque Sandstone (Formation) and the off-shore mudrocks of the Rio Salado Tongue of the lower Mancos Shale (Fig. 2) (Hook, Molenaar & Cobban, 1983; McLellan et al., 1983a; Molenaar et al., 2002). The Moreno Hill is regionally overlain by a temporal unconformity (Cenozoic sediments and volcanics) and is down-cut by the Eocene Baca and Miocene Fence Lake Formations (locally variable) (McLellan et al., 1982, 1983a; Roybal, 1982; Campbell, 1984, 1989; Campbell & Roybal, 1984; Cook & Arkell, 1987). Based on current mapping, the three unnamed members (lower, middle, and upper) of the Moreno Hill constitute a clastic wedge (Anderson, 1981; Roybal, 1982; McLellan et al., 1983a; Campbell, 1984, 1989; Campbell & Roybal, 1984; McLellan, Landis & Biewick, 1984; Landis et al., 1985; Cook & Arkell, 1987). The first formal lithostratigraphic identification of the Moreno Hill Formation was by McLellan et al. (1983a), with subsequent revisions by Campbell (1984), McLellan, Landis & Biewick (1984) and Hoffman (1996), which provided further sedimentological context for the Moreno Hill including depo-center characterization and paleoclimatic proxies (humidity levels). Most research has focused on regional correlation and economic potential of three key coal seams, the lower Antelope, the medial Cerro Prieto, and the upper Rabbit, all within the lower Moreno Hill (Anderson, 1982a, 1982b, 1983; McLellan et al., 1983a; Campbell, 1984; Campbell & Roybal, 1984; Cook & Arkell, 1987; Hoffman, 1994, 1996). Current biostratigraphic age control for the Atarque Sandstone is based on the local occurrence of the range zones of *Collignoniceras woollgari* and *Mammities nodosoides*. The maximum age is bounded by the middle Turonian ammonites *Collignoniceras woollgari woollgari* and *Mammities nodosoides* and bivalve *Mytiloides mytiloides* within the underlying Rio Salado Tongue of the Mancos Shale (Cobban & Hook, 1983) and by the co-occurrence of *Collignoniceras woollgari woollgari* with *Mytiloides labiatus* (Wolfe & Kirkland, 1998, p. 304) or *Mytiloides mytiloides* (Kirkland, Smith & Wolfe, 2005, p. 89) in the Atarque Sandstone. The D-Cross (Pescado) Tongue and Gallup Sandstone (correlative to the lower member of the Moreno Hill Formation) in the Pescado Creek, upper Nutria and Gallup areas contain the oysters *Cameleolopha lugubris* and *C. bellaplicata* as do the lower Juana Lopez beds of the D-Cross tongue at Bull Gap; radiometrically dated bentonite beds within these zones indicate an age of ~90 Ma (Wolfe & Kirkland, 1998; Molenaar et al., 2002; Hook & Cobban, 2011, 2012, 2013). Regional correlations for the Moreno Hill have also been assessed via sequence- and lithostratigraphic linkages to the north-eastern Zuni Basin, with emplacement of sediment roughly during the regional R-1 phase of the Greenhorn Cycle and during the New Mexico-specific T-2—R-2 Carlile Cycle (early Niobrara Cyclothem equivalent) (Hook, Molenaar & Cobban, 1983; Molenaar, 1983a, 1983b; Wolfe, 1989; Wolfe & Kirkland, 1998; Kauffman et al., 1993; Molenaar et al., 2002; Hook, 2010; Hook & Cobban, 2011).

**Figure 3:**
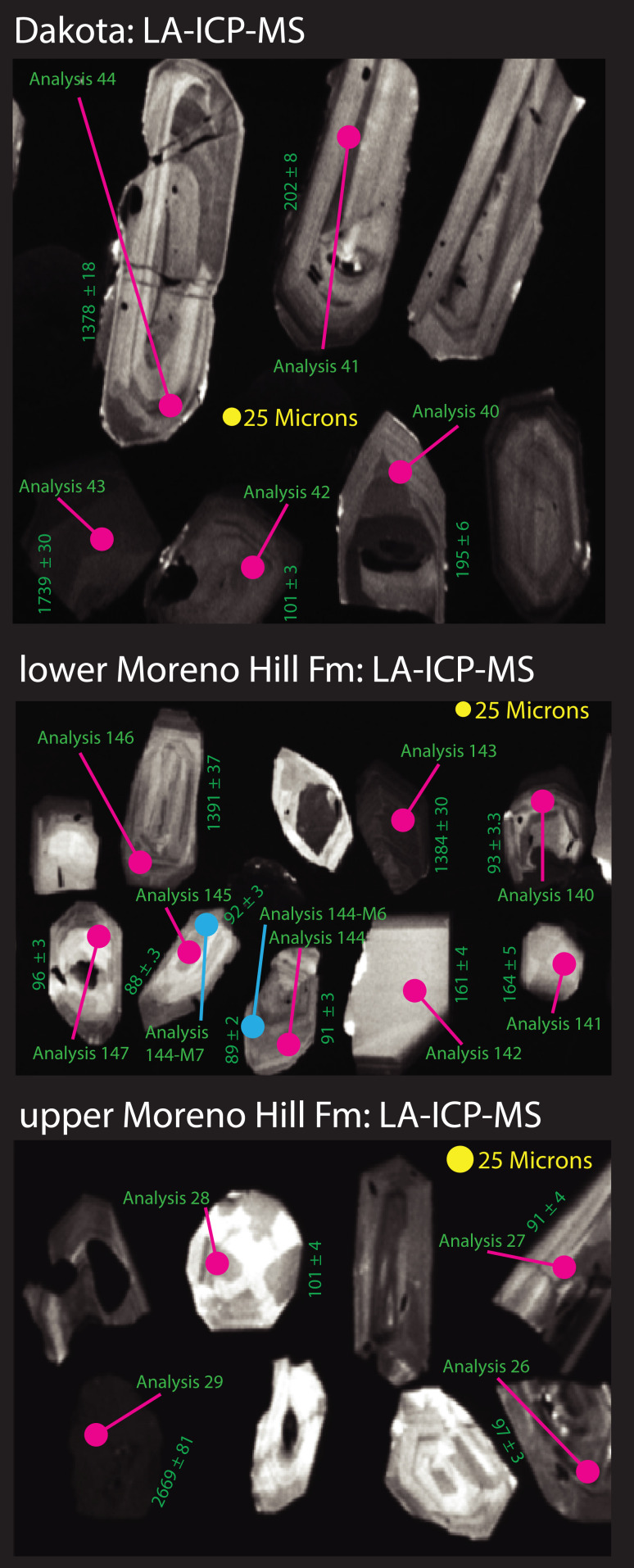
Cathodoluminescence images of zircons. Grains selected for LA-ICPMS & CA-TIMS are shown with LA-ICP-MS spots and analysis labels.

## Methods

All Moreno Hill samples were collected from the eastern slopes of Santa Rita Mesa at Cox Ranch, located north of Quemado (Salt Lake Coal Field), west-central New Mexico (BLM Permit NM17-02S). Three key stratigraphic zones were selected based on stratigraphic position or proximity to known fossil horizons (Fig. 2). Specifically, bulk samples were collected from the contact with the underlying Atarque (basal Moreno Hill (Cox 2), along with the middle (Cox 3) and uppermost (Cox 4) bedded sandstone strata of the Moreno Hill Formation. A fourth sample was collected near Zuni Salt Lake from the underlying Dakota Sandstone and is also presented herein (whilst Carpenter (2014) notes that “Naturita Sandstone” is a more apt name for this unit on the Colorado Plateau we prefer “Dakota Sandstone” to facilitate reference to previous research). All samples collected were fresh and unweathered; at least 0.5–1.0 m of the exterior surface of the rock was removed from the outcrop face before collecting due to surficial weathering. Two gallon-sized bags of sandstone were collected at each site (Dakota Sandstone, Cox 2, 3, and 4) and processed accordingly to the techniques set forth by the Central Analytical Facility at Stellenbosch University including crushing, milling, panning, Frantz magnetic separation, and density liquid separation. Thereafter, analysis of the recovered zircon grains was undertaken via standard methods of Boise State University’s Isotope Geology Laboratory (Boise, Idaho, USA) as noted in several other studies (Bold, 2016; MacNaughton et al., 2016; Normore et al., 2018; Tucker et al., 2020).

### LA-ICP-MS methods

Zircon grains were annealed at 900 °C for 60 h in a muffle furnace, and randomly selected grains were mounted in epoxy and polished until their centers were exposed (Fig. 3). Cathodoluminescence (CL) images were obtained with a JEOL JSM-300 scanning electron microscope and Gatan MiniCL. Zircons were analyzed by Laser Ablation-Inductively Coupled Plasma-Mass Spectrometry (LA-ICP-MS) using a ThermoElectron X-Series II quadrupole ICPMS and New Wave Research UP-213 Nd:YAG UV (213 nm) laser ablation system. In-house analytical protocols, standard materials, and data reduction software were used for acquisition and calibration of U-Pb dates and a suite of high field strength elements (HFSE) and rare earth elements (REE). Zircons were ablated with a laser spot of 25 µm wide using fluence and pulse rates of 5 J/cm^2^ and 5 Hz, respectively, during a 45-s analysis (15-s gas blank, 30-s ablation) that excavated a pit ~15 µm deep. Ablated material was carried by a 1.2 L/min He gas stream to the nebulizer flow of the plasma. Quadrupole dwell times were 5 ms for Si and Zr, 200 ms for ^49^Ti and ^207^Pb, 80 ms for ^206^Pb, 40 ms for ^202^Hg, ^204^Pb, ^208^Pb, ^232^Th, and ^238^U and 10 ms for all other HFSE and REE; total sweep duration is 950 ms. Background count rates for each analyte were obtained prior to each spot analysis and subtracted from the raw count rate for each analyte. For concentration calculations, background-subtracted count rates for each analyte were internally normalized to ^29^Si and calibrated with respect to NIST SRM-610 and -612 glasses as the primary standards. Ablations pits that appear to have intersected glass or mineral inclusions were identified based on Ti and P signal excursions, and associated sweeps were discarded. U-Pb dates from these analyses are considered valid if the U-Pb ratios appear to have been unaffected by the inclusions. Signals at mass 204 were normally indistinguishable from zero following subtraction of mercury backgrounds measured during the gas blank (<1,000 cps ^202^Hg), and thus dates are reported without common Pb correction. Rare analyses that appear contaminated by common Pb were rejected based on mass 204 greater than baseline. Temperature was calculated from the Ti-in-zircon thermometer (Watson, Wark & Thomas, 2006). Because there are no constraints on the activity of TiO_2_, an average value in crustal rocks of 0.8 was used.

Data were collected in three experiments in July 2020. For U-Pb and ^207^Pb/^206^Pb dates, instrumental fractionation of the background-subtracted ratios was corrected and dates were calibrated with respect to interspersed measurements of zircon standards and reference materials. The primary standard Plešovice zircon (Sláma et al., 2008) was used to monitor time-dependent instrumental fractionation based on two analyses for every 10 analyses of unknown zircons.

Radiogenic isotope ratio and age error propagation for all analyses include uncertainty contributions from counting statistics and background subtraction. The standard calibration uncertainty for U/Pb is the local standard deviation of the polynomial fit to the fractionation factor of Plešovice vs time and for ^207^Pb/^206^Pb is the standard error of the mean of the fractionation factor of Plešovice. These uncertainties are 0.6–1.0% (2s) for ^206^Pb/^238^U and 0.2–0.4% (2s) for ^207^Pb/^206^Pb. Age interpretations are based on ^207^Pb/^206^Pb dates for analyses with ^207^Pb/^206^Pb and ^206^Pb/^238^U dates >1,000 Ma. Otherwise, interpretations are based on ^206^Pb/^238^U dates. Analyses with ^206^Pb/^238^U dates >1,000 Ma and >10% positive discordance or >5% negative discordance are not considered. Errors on the dates are given at 2 sigma.

### CA-TIMS U-Pb geochronology methods

U-Pb dates were obtained by the Chemical Abrasion Isotope Dilution Thermal Ionization Mass Spectrometry (CA-TIMS) method from analyses composed of single zircon grains (Table 1), modified after Mattinson (2005). Zircon was removed from the epoxy mounts for dating based on CL images and LA-ICP-MS dates. In two samples, grains that yielded the five youngest LA-ICP-MS dates from an initial round were analyzed in a second round. Dates from both rounds agreed in most cases. Grains were selected for CA-TIMS from this population. For a third sample, the grains were too small to permit a second round of analysis.

**Table 1:** U/Pb CA-TIMS isotopic data Pb/U. MDAs via youthful detrital zircons.

Sample	LA-ICPMS							Radiogenic Isotope Ratios								Isotopic Dates					
		$\rm \frac{Th}{U}$	^206^Pb* × 10^−13^ mol	mol % ^206^Pb*	$\rm \frac {Pb}{Pb_c}$*	Pb_c_ (pg)	$\frac{\displaystyle\bf{^{206}Pb}}{\displaystyle{\bf{^{204}Pb}}}$	${\displaystyle\bf{^{208}Pb}\over\displaystyle{\bf{^{206}Pb}}}$	$\displaystyle\bf{^{207}Pb}\over\displaystyle{^{\bf 206}\bf Pb}$	% err	$\displaystyle\bf{^{207}Pb}\over\displaystyle{\bf{^{235}U}}$	% err	$\frac{\displaystyle\bf{^{206}Pb}}{\displaystyle{\bf{^{238}U}}}$	% err	corr. coef.	$\displaystyle{\bf{^{207}Pb}}\over\displaystyle{\bf{^{206}Pb}}$	±	$\displaystyle{\bf{^{207}Pb}}\over\displaystyle{\bf{^{235}U}}$	±	$\displaystyle{\bf{^{206}Pb}}\over\displaystyle{\bf{^{238}U}}$	±
(a)	label	(b)	(c)	(c)	(c)	(c)	(d)	(e)	(e)	(f)	(e)	(f)	(e)	(f)		(g)	(f)	(g)	(f)	(g)	(f)
MHL-Cox 2																					
z1	144	0.971	0.2723	99.25%	44.5	0.17	2392	0.311	0.047845	0.303	0.094027	0.321	0.014260	0.053	0.422	90.54	7.18	91.25	0.28	91.275	0.048
z2	145	0.513	0.2190	99.45%	54.5	0.10	3279	0.164	0.048009	0.226	0.093912	0.244	0.014194	0.045	0.496	98.63	5.33	91.14	0.21	90.855	0.040
MHM-Cox 3																					
z1	262	0.438	0.3606	99.52%	61.7	0.14	3789	0.140	0.048001	0.198	0.102478	0.210	0.015491	0.051	0.343	98.25	4.69	99.06	0.20	99.095	0.051
z2	263	1.472	0.2384	99.49%	74.4	0.10	3566	0.471	0.047968	0.229	0.094457	0.248	0,014288	0.047	0.483	96.65	5.43	91.65	0.22	91.454	0.043
MHU-Cox 4																					
z2	33	0.503	0.1096	98.88%	26.6	0.10	1618	0.161	0.048049	1.003	0.097621	1.064	0.014742	0.152	0.464	100.64	23.71	94.58	0.96	94.338	0.142
z1a	17	0.562	0.0828	98.15%	16.2	0.13	978	0.180	0.047993	0.755	0.091585	0.796	0.013847	0.084	0.525	97.87	17.86	88.98	0.68	88.648	0.074
z1b	17	0.627	0.0767	89.79%	2.7	0.72	177	0.201	0.048354	2.643	0.092014	2.779	0.013808	0.326	0.465	115.55	62.31	89.38	2.38	88.401	0.286

**Notes:**

(a) z1, z2, etc. are labels for analyses composed of single zircon grains that were annealed and chemically abraded (Mattinson, 2005). z1a and z1b are fragments from the same grain.

(b) Model Th/U ratio calculated from radiogenic 208Pb/206Pb ratio and 207Pb/235U date.

(c) Pb* and Pbc are radiogenic and common Pb, respectively. mol % ^206^Pb* is with respect to radiogenic and blank Pb.

(d) Measured ratio corrected for spike and fractionation only. Fractionation correction for single-collector Daly analyses is based on measurement of 202Pb/205Pb in the EARTHTIME ET2535 tracer solution.

(e) Corrected for fractionation and spike. Common Pb in zircon analyses is assigned to procedural blank with composition of 206Pb/204Pb = 18.04 ± 0.61%; 207Pb/204Pb = 15.54 ± 0.52%; 208Pb/204Pb = 37.69 ± 0.63% (1 sigma). 206Pb/238U and 207Pb/206Pb ratios corrected for initial disequilibrium in 230Th/238U using a D(Th/U) of 0.20 ± 0.05 (1 sigma).

(f) Errors are 2 sigma, propagated using algorithms of Schmitz & Schoene (2007) and Crowley, Schoene & Bowring (2007).

(g) Calculations based on the decay constants of Jaffey et al. (1971). 206Pb/238U and 207Pb/206Pb dates corrected for initial disequilibrium in 230Th/238U using a D(Th/U) of 0.20 ± 0.05 (1 sigma).

Zircon was put into 3 ml Teflon PFA beakers and loaded into 300 ml Teflon PFA microcapsules. Fifteen microcapsules were placed in a large-capacity Parr vessel and the zircon partially dissolved in 120 ml of 29 M HF for 12 h at 190 °C. Zircon was returned to 3 ml Teflon PFA beakers, HF was removed, and zircon was immersed in 3.5 M HNO_3_, ultrasonically cleaned for an hour, and fluxed on a hotplate at 80 °C for an hour. The HNO_3_ was removed and zircon was rinsed twice in ultrapure H_2_O before being reloaded into the 300 ml Teflon PFA microcapsules (rinsed and fluxed in 6 M HCl during sonication and washing of the zircon) and spiked with the EARTHTIME mixed ^233^U-^235^U-^202^Pb-^205^Pb tracer solution (ET2535). Zircon was dissolved in Parr vessels in 120 ml of 29 M HF with a trace of 3.5 M HNO_3_ at 220 °C for 48 h, dried to fluorides, and re-dissolved in 6 M HCl at 180 °C overnight. U and Pb were separated from the zircon matrix using an HCl-based anion-exchange chromatographic procedure (Krogh, 1973), eluted together and dried with 2 µl of 0.05 N H_3_PO_4_.

Pb and U were loaded on a single outgassed Re filament in 5 µl of a silica-gel/phosphoric acid mixture (Gerstenberger & Haase, 1997), and U and Pb isotopic measurements made on a GV Isoprobe-T multicollector thermal ionization mass spectrometer equipped with an ion-counting Daly detector. Pb isotopes were measured by peak-jumping all isotopes on the Daly detector for 160 cycles and corrected for mass fractionation using the known ^202^Pb/^205^Pb ratio of the ET2535 tracer solution. Transitory isobaric interferences due to high-molecular-weight organics, particularly on ^204^Pb and ^207^Pb, disappeared within approximately 30 cycles, while ionization efficiency averaged 10^4^ cps/pg of each Pb isotope. Linearity (to ≥ 1.4 × 10^6^ cps) and the associated deadtime correction of the Daly detector were determined by analysis of NBS982. Uranium was analyzed as UO_2_^+^ ions in static Faraday mode on 10^12^ ohm resistors for 300 cycles, and corrected for isobaric interference of ^233^U^18^O^16^O on ^235^U^16^O^16^O with an ^18^O/^16^O of 0.00206. Ionization efficiency averaged 20 mV/ng of each U isotope. U mass fractionation was corrected using the known ^233^U/^235^U ratio of the ET2535 tracer solution.

U-Pb dates and uncertainties were calculated using the algorithms of Schmitz & Schoene (2007), calibration of ET2535 tracer solution (Condon et al., 2015) of ^235^U/^205^Pb = 100.233, ^233^U/^235^U = 0.99506, ^205^Pb/^204^Pb = 8474, and ^202^Pb/^205^Pb = 0.99924, U decay constants recommended by Jaffey et al. (1971), and ^238^U/^235^U of 137.818 (Hiess et al., 2012). The ^206^Pb/^238^U ratios and dates were corrected for initial ^230^Th disequilibrium using D_Th/U_ = 0.2 ± 0.1 (2 sigma) and the algorithms of Crowley, Schoene & Bowring (2007), resulting in an increase in the ^206^Pb/^238^U dates of ~0.09 Ma. All common Pb in analyses was attributed to laboratory blank and subtracted based on the measured laboratory Pb isotopic composition and associated uncertainty. U blanks are estimated at 0.013 pg.

A weighted mean ^206^Pb/^238^U date is calculated from equivalent dates (probability of fit > 0.05) using Isoplot 3.0 (Ludwig, 2003). The error is given as ±x/y/z, where x is the internal error based on analytical uncertainties only, including counting statistics, subtraction of tracer solution, and blank and initial common Pb subtraction, y includes the tracer calibration uncertainty propagated in quadrature, and z includes the ^238^U decay constant uncertainty propagated in quadrature. Internal error should be considered when comparing our date with ^206^Pb/^238^U dates from other laboratories that used the same tracer solution or a tracer solution that was cross-calibrated using EARTHTIME gravimetric standards. Error including the uncertainty in the tracer calibration should be considered when comparing our date with those derived from other geochronological methods using the U-Pb decay scheme (e.g., laser ablation ICPMS). Error including uncertainties in the tracer calibration and ^238^U decay constant (Jaffey et al., 1971) should be considered when comparing our date with those derived from other decay schemes (e.g., ^40^Ar/^39^Ar, ^187^Re-^187^Os). Errors are at 2 sigma.

## Results

### CA-TIMS

Results from CA-TIMS dating are used to establish MDAs for the Moreno Hill Formation (Table 1). Two grains from the lower Moreno Hill Formation (Cox 2) yield CA-TIMS dates of 91.275 ± 0.048 and 90.855 ± 0.040 Ma, indicating that deposition occurred after 90.9 Ma. Two grains from the middle Moreno Hill Formation (Cox 3) yield dates of 99.095 ± 0.051 and 91.454 ± 0.043 Ma. These dates are older than the MDA from the underlying sample, and thus the grains are not close in age to sedimentation. For the upper Moreno Hill Formation (Cox 4) two grains were analyzed by CA-TIMS. One yields a date of 94.338 ± 0.142 Ma that is older than the MDA from the underlying lower Moreno Hill Formation, and thus the grain is not close in age to sedimentation. The other was broken into two fragments that were analyzed separately and yield equivalent dates of 88.648 ± 0.074 and 88.401 ± 0.286 Ma, with a weighted mean of 88.632 ± 0.072/0.084/0.127 Ma (MSWD = 2.8, probability of fit = 0.09) (Table 1). This is taken as the MDA, indicating deposition was after 88.6 Ma. Based on the assumption that the MDAs from the lower and upper Moreno Hill Formation are close in age to deposition, the formation is interpreted as being deposited between the Late Turonian and earliest Coniacian.

### LA-ICP-MS

**Figure 4:**
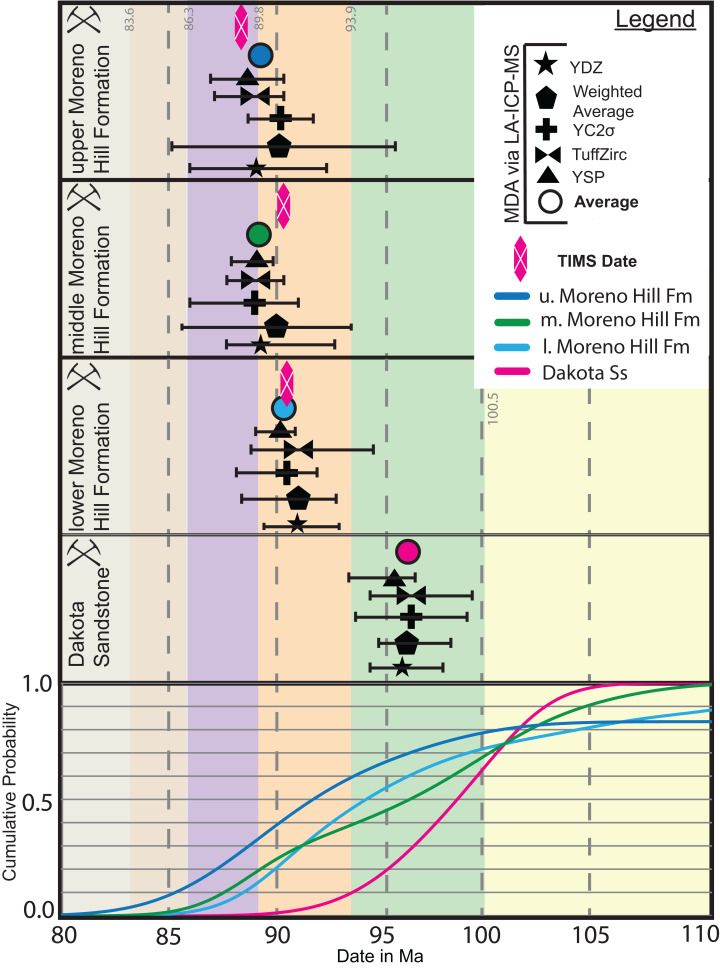
LA-ICPMS MDA. Temporal relationship of the five (5) metrics (YDZ, Weighted Average, YC2σ, TuffZirc, and YSP) utilized within this study, within stratigraphic order. YDZ, Weighted Average and TuffZirc were run via Isoplot 4.15 (Ludwig, 2003), YC2σ via Age Pick Program (2010) G. Gehrels 1 September 2009, Arizona Laserchron Center, and YSP via Herriott et al. (2019). Also displayed is the K–S test to show the variable weak genetic relationship across the samples across the stratigraphic section. K–S analysis after Barbeau et al. (2009b).

The four samples (Dakota Sandstone and lower, middle, and upper Moreno Hill Formation) contained a broad spectrum of detrital zircon. Individual grains range from well-faceted euhedral, to somewhat rounded or minorly cracked, to fractured, rounded, or fragmented. All of the grains (save for a minor few) have oscillatory and sector zoning indicative of igneous growth (Fig. 3). Many grains have distinct cores. Zircons in the Dakota Sandstone are more commonly round, indicative of transport, compared with the well-faceted zircon that is common in the lower to middle Moreno Hill. Based on the recent work of Coutts, Matthews & Hubbard (2019), Herriott et al. (2019), and Beveridge, Roberts & Titus (2020), our study also ran an additional filter based on Tucker et al. (2013, 2016, 2020), which omits analyses with a greater than 5% (at 2σ analytical uncertainty) discordance based on the ^207^Pb/^235^U and ^206^Pb/^238^U ratios. The 5% filter is more rigorous than Tucker et al. (2013, 2016), which utilized 10–15%, based on the recent results by Beveridge, Roberts & Titus (2020). For LA-ICPMS-based MDAs, we utilized the following five analyses for sample sets ranging from 30 to 100 grains: YDZ (*n* = 6) (Youngest Detrital Zircon); YC2σ (*n* = 6) (Youngest Cluster of grains with overlapping 2σ uncertainty); Weighted Average (*n* = 6); and TuffZirc (*n* = 6); with the new addition of YSP (*n* > 6) (Youngest Statistical Population) (Ludwig, 2003; Coutts, Matthews & Hubbard, 2019; Herriott et al., 2019). Results are described herein and depicted in Fig. 4.

### Dakota sandstone

In agreement with Pike (1947), Wolfe (1989) and more recently Carpenter (2014), and based on regional characterization of the trough-bedded alluvial sandstone, this sample was recovered from the laterally extensive lower “Dakota alluvial unit” or “main body of the Dakota Sandstone” (McLellan et al., 1983b). We do not, however, recognize the “Cliff Dwellers Sandstone” nomenclature for this unit (Wolfe, 1989). The Dakota Sandstone has the widest spectrum of dates and grain morphologies. Of the 97 grains ablated (86 included), 31 are Precambrian, four are Paleozoic, and 51 grains are Mesozoic. These youngest date signatures are YDZ at 96.13 +1.6/−2.0 Ma, Weighted Average at 96.5 ± 1.6 Ma, YC2σ at 96.5 ± 2.5 Ma (MSWD = 1.2), TuffZirc date at 96.3 +3.2/−1.8 Ma, and YSP 95.2 ± 0 Ma (MSWD = 0.96) (Fig. 4; Table 2). The mean of the youngest date signatures is 96.1 Ma (Table 2; Table S1). Based on this date we suspect that this lower “Dakota sandstone” is equivalent to the basal Naturita Sandstone in Southern Utah (Barclay et al., 2015; Laurin et al., 2019; Tucker et al., 2020).

**Table 2:** Comparison of different LA-ICPMS-MDA metrics. Comparison of five (5) different metrics utilized within this study to interpret the Maximum Depositional Age (MDAs) for each detrital sample (modified from Tucker et al. (2013, 2016), Coutts, Matthews & Hubbard (2019), Herriott et al. (2019), Tucker et al. (2020)). YDZ, Weighted Average and TuffZirc were run via Isoplot 4.15 (Ludwig, 2003), YC2σ via Age Pick Program (2010) G. Gehrels 1 September 2009, Arizona Laserchron Center, and YSP via Herriott et al. (2019).

Analysis		Dakota Sandstone	Lower Moreno Hill	Middle Moreno Hill	Upper Moreno Hill
YDZ (*N* = 6)	Final Age	96.12	91.16	89.5	89.3
	Range	+1.6/−2.0	+2.5/−2.1	+3.1/−1.8	+3.4/−2.4
	Confidence	95%	95%	95%	95%
WEIGHTED AVERAGE (*N* = 6)	Final Age	96.5 (±1.6)	91.4 (±2.9)	90.2 (±3.7)	90.5 (±4.9)
	Confidence	95.0%	95.0%	95.0%	95.0%
	Rejection	0	0	0	0
	MSWD	1.2	4.2	7.9	5.1
	Probability	0.30	0.0	0.0	0.0
YC2σ (*N* = 6)	Final Age	96.5 ± 2.5 (2.6%)	90.2 ± 2.3 (2.5%)	89.1 ± 2.5 (2.8%)	90.5 ± 3.3 (3.6%)
	Weighted Mean Age	96.5 ± 2.3 (2.4%)	90.2 ± 2.1 (2.3%)	89.1 ± 2.2 (2.5%)	90.5 ± 3.1 (3.4%)
	Systematic Error	1.1%	1.1%	1.1%	1.1%
	MSWD	0.3	2.0	0.0	1.3
TUFF ZIRC (*N* = 6)	Age	96.32	91.69	89.36	89.01
		+3.19/−1.81	+3.02/−2.42	+1.49/−1.79	+1.69/−1.81
	Confidence	96.6%	93.8	87.6%	87.6%
	Group Size	6 of 6	5 of 6	4 of 6	4 of 6
YSP	Final Age	95.19	90.21	88.67	88.82
	Error (±2σ)	±0 (1.26)	±1.2 (1.65)	±1.1 (1.52)	±1.7 (2.1)
	MSDW	0.96	1.19	1.38	0.94
	PoF	0.46	0.312	0.247	0.418
Average		96.1 Ma	90.9 Ma	89.3 Ma	89.6 Ma

### Lower Moreno Hill Formation

Of the 101 grains ablated (88 included), 60 are Precambrian, Paleozoic grains are absent, and 28 are Mesozoic. The youngest date signatures are YDZ at 91.16 +2.5/−2.1 Ma, Weighted Average at 91.4 + 2.9 Ma, YC2σ at 90.2 ± 2.5 Ma, TuffZirc date at 91.7 +3.0/−2.4 Ma, YSP at 90.2 ± 1.2 Ma (MSWD = 1.19) (Fig. 4; Table 2). The mean of the youngest date signatures is 90.9 Ma (Table 2; Table S1).

### Middle Moreno Hill Formation

Of the 99 grains ablated (94 included), 57 are Precambrian; Paleozoic grains are absent, and 37 grains are Mesozoic. The youngest grain signatures are YDZ at 89.5 +3.4/−2.4 Ma, Weighted Average at 90.2 ± 3.7 Ma, YC2σ at 89.1 ± 2.2 Ma, TuffZirc date at 89.4 +1.5/−1.8 Ma, YSP at 88.67 ± 1.1 Ma (MSWD = 1.38) (Fig. 4; Table 2). The mean of the youngest date signatures is 89.3 Ma (Table 2; Table S1).

### Upper Moreno Hill Formation

Of the 24 grains ablated (16 included), three grains are Precambrian, one is Paleozoic, and 12 are Mesozoic. The youngest date signatures are YDZ at 89.3 +3.4/−2.4 Ma, Weighted Average at 90.5 ± 4.9 Ma, YC2σ at 90.5 ± 3.3 Ma, TuffZirc date at 89.0 +1.7/−1.8 Ma, and YSP at 88.82 ± 1.7 Ma (MSWD = 0.94) (Fig. 4; Table 2). The mean of the youngest date signatures is 89.6 Ma (Table 2; Table S1).

### Detrital populations: precambrian & phanerozoic-paleozoic

**Figure 5:**
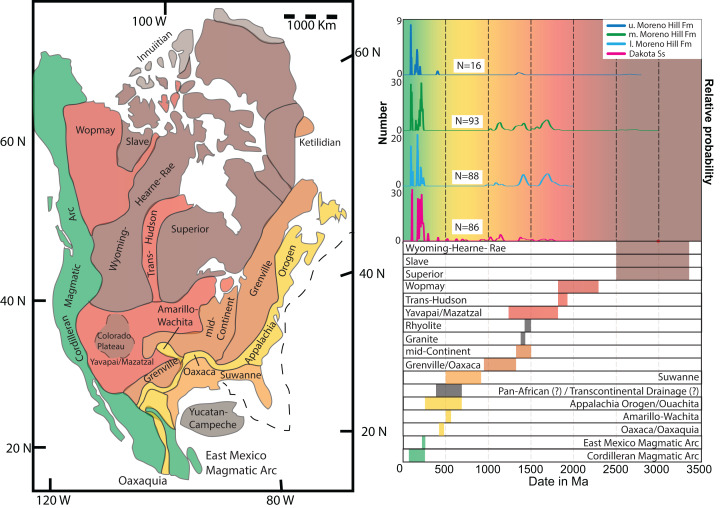
Detrital zircon provenance. Combined possible crustal provenances and age-distribution curve for all analyzed detrital zircons from the Dakota Sandstone and the three Moreno Hill Formation samples. North American map displays likely crustal provenances for each major and minor detrital zircon age population reworked within the Cordilleran retroarc foreland basin system. Successive zircon age populations (displayed in the age-distribution curve on the right; and are displayed with color corresponding to the select crustal provinces). Modified and adapted from Dickinson & Gehrels (2009a) and Laskowski, DeCelles & Gehrels (2013); and references therein. Age placement based on Cohen et al. (2013, v2019/05). Figure also modified from Tucker et al. (2020). Probability density plots via Isoplot 4.15 (Ludwig, 2003).

This study builds upon well-documented tectonic reconstructions and source terranes (Fig. 5) (Willis, 1999; Dickinson & Gehrels, 2003, 2008, 2009a; 2009b; DeCelles, 2004; Lawton & Bradford, 2011; Laskowski, DeCelles & Gehrels, 2013), which allow for reliable linkages. The Dakota Sandstone sample displays a diverse assemblage of grain ages and populations. In contrast, zircons from the Moreno Hill Formation are either Precambrian *n* > 1.0 Ga or Mesozoic (*n* < 251 Ma). All samples contain individual grains or minor populations that are >2.0 Ga and are likely reworked continental fragments (Gehrels et al., 1995; Linde et al., 2016; Tucker et al., 2020). Large populations of grains between 1.9 and 1.5 Ga were identified in all samples. As a result of multi-phased sedimentary recycling, these detrital zircons can be meaningfully associated with (1) the Trans-Hudson and Wopmay orogenies (2.0–1.5 Ga); (2) the Yavapai/Mazatzal terranes, and; (3) the Grenville terrane (1.7–1.0 Ga) (Gehrels et al., 1995; Van Schmus, Bickford & Turek, 1996; Whitmeyer & Karlstrom, 2007). Due to the distance of the Sevier (west) and Maria (south-southwest) Fold and Thrust Belts, grains between 1.7 and 1.2 Ga are likely from uplifted basement (Yavapai/Mazatzal). Therefore this study is in agreement with regional studies that the most likely source terrane for the above-mentioned grains is the Mogollon Highlands (Barth et al., 2004; Dickinson & Gehrels, 2008; Salem, 2009; Lawton & Bradford, 2011; Laskowski, DeCelles & Gehrels, 2013; Szwarc et al., 2015). Our study also recognized the possibility, though less likely, that source terranes include but are not limited to (1) Antarctica; (2) Australia; (3) Africa; or even yet to be identified proto-Rodinian terranes (Gehrels & Stewart, 1998; Laskowski, DeCelles & Gehrels, 2013; Linde et al., 2016). Of the four samples included in this study, only the Dakota Sandstone contains a population of Paleozoic grains (*n* = 4). This may reflect a bias in sampling or more likely that sources such as the Amarillo-Wichita to Appalachian Orogeny as noted by Laskowski, DeCelles & Gehrels (2013) did not contribute much into this area. In any event, dates from this period in North America’s tectonic history are distinctly absent within the Moreno Hill Formation (Fig. 5).

### Detrital populations: phanerozoic-mesozoic

**Figure 6:**
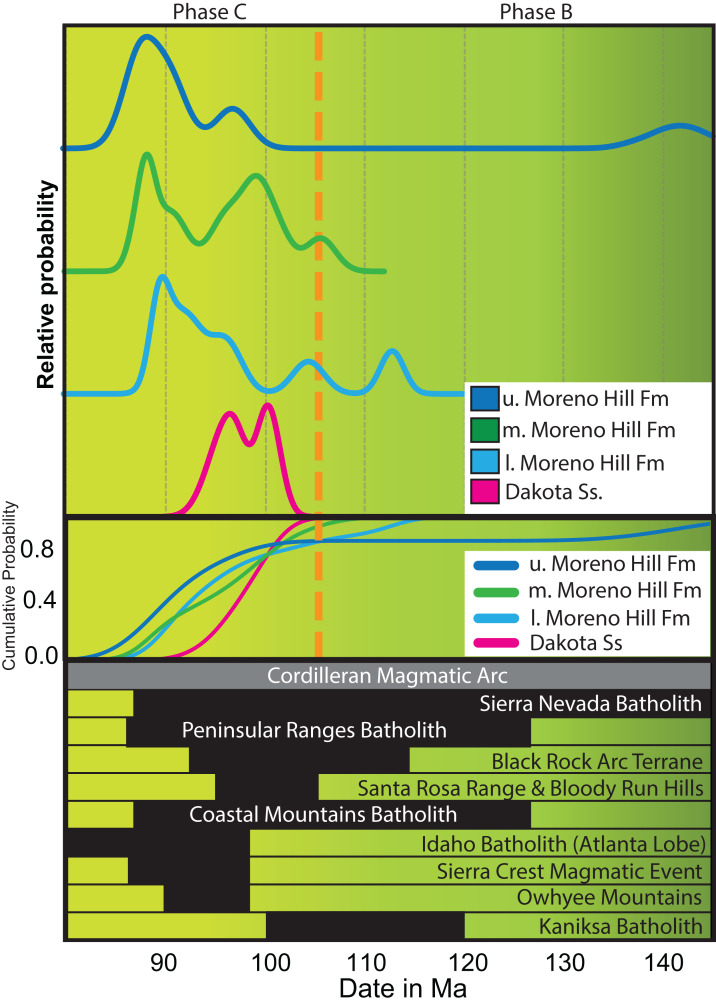
Youthful detrital zircon provenance. Plausible sources for the most youthful detrital zircon populations recovered within this study. Samples fall within phase B and C, with the most youthful populations in phase C. Information of various inliers recovered from Dickinson & Gehrels (2009b); Gaschnig et al. (2010); Lawton, Hunt & Gehrels (2010); Hunt et al. (2011); Laskowski, DeCelles & Gehrels (2013); DeCelles & Graham (2015); Linde et al. (2016); Sauer et al. (2017); Brown, Hart & Stuck (2018); and references therein. Age placement based on Cohen et al. (2013, v2019/05). K–S analysis after Barbeau et al. (2009b).

We describe the Mesozoic detrital zircon populations from the Dakota Sandstone and the overlying Moreno Hill Formation separately. For both, we describe the youngest populations in accordance with DeCelles (2004): Phases A (160–140 Ma); B (140–105 Ma) and C (105–80 Ma) (Fig. 6) (DeCelles & Graham, 2015). In the Dakota Sandstone, the majority of dates are Triassic (*n* = 24) and Jurassic (*n* = 20) with the remaining few (*n* = 9) being Cretaceous. Specific Triassic—Jurassic sediment source terranes, which rely on well-understood southern Cordilleran orogenic systems that were active between 260 and 145 Ma include the (1) East Mexico Arc; (2) Mojave Desert, and; (3) Wallowa/Olds Ferry terranes; along with early phases of the (4) Western Coast Plutonic Complex; (5) Sierra Nevada Batholith; and (6) Omineca Belt (Gehrels & Stewart, 1998; Gehrels et al., 2009; LaMaskin, 2012; Gehrels & Pecha, 2014; Gaschnig et al., 2017; Quinn et al., 2018). All of these grain populations can also be linked to heavily-reworked multi-generational recycling of sedimentary blankets lying east of the Sevier Highlands and north of the Mogollon Highlands, potentially including proximal units underlying the sub-Dakota Sandstone angular unconformity (Wolfe, 1989; Molenaar et al., 2002; Dickinson & Gehrels, 2003, 2009a, 2009b; Lawton, Pollock & Robinson, 2003; DeCelles, 2004; Laskowski, DeCelles & Gehrels, 2013; Gehrels & Pecha, 2014).

Other than the notable Precambrian signatures, all four samples contain distinct Late Jurassic – Cretaceous multi-grain populations with the majority within phases A (160–140 Ma) and B (140–105 Ma) and the most youthful populations falling within Phase C (105–80 Ma) (DeCelles & Graham, 2015). All samples assessed in this study contain multi-grain populations within Phase C, which we link to the westerly lying Cordilleran Arc; more specifically, the southern portion of the Sierra Nevada Batholith, the northern portion of the Peninsular Ranges Batholith, and minor volcanism near the Maria Fold and Thrust Belt (DeCelles, 2004; Dickinson, 2008; Busby, Busby & Azor, 2012; Hildebrand & Whalen, 2014; LaMaskin, 2012; Szwarc et al., 2015; Pecha et al., 2018; Lawton, 2019; DeGraaff Surpless et al., 2019). Shorter-lived and more-localized volcanic events within Phases B and C that could have been contributors include the (1) Black Rock Arc Terrane (114–103 Ma); (2) Sierra Crest Magmatic Event (98–86 Ma); (3) Santa Rosa Range and the Bloody Run Hills (110–85 Ma); and (4) Soldier and Cathedral peak plutons (Fig. 6) (Dickinson & Gehrels, 2008; Lawton, Hunt & Gehrels, 2010; Hunt et al., 2011; Laskowski, DeCelles & Gehrels, 2013; Cao et al., 2015, Fig. 15, p. 316, cycles 1, 2 and early Phase 3; Szwarc et al., 2015; Brown, Hart & Stuck, 2018; Pecha et al., 2018; Tucker et al., 2020). Within the southwestern portion of the Arc, possible Phase C contributors of the most youthful zircon populations include the southeastern zone of the Sierra Nevada Batholith (less likely Central Sierra Nevada Batholith) and the northeastern zone of the Peninsular Ranges Batholith (Fig. 6) (Hildebrand & Whalen, 2014; DeCelles & Graham, 2015; Yonkee & Weil, 2015). Cretaceous grain populations could yet be linked to the Mogollon Highlands where Pike (1947) noted sedimentary rocks (with interbedded volcanics) indicated by fossil content to be “Benton-aged” near Deer Creek, Arizona.

Lastly, in light of recent literature documenting highly variable grain sorting and emplacement histories, other much less-probable but potential sources include (1) Owhyee Mountains; (2) Atlanta Lobe of the Idaho Batholith; and (3) Eastern Coast Plutonic Complex (Parrish, Gaynor & Swift, 1984; Slingerland et al., 1996; Gaschnig et al., 2010; Fricke, Foreman & Sewall, 2010; Hay & Floegel, 2012; Laskowski, DeCelles & Gehrels, 2013; Finzel, 2017; Sauer et al., 2017; Brown, Hart & Stuck, 2018; Quinn et al., 2018).

### K–S analysis

**Figure 7:**
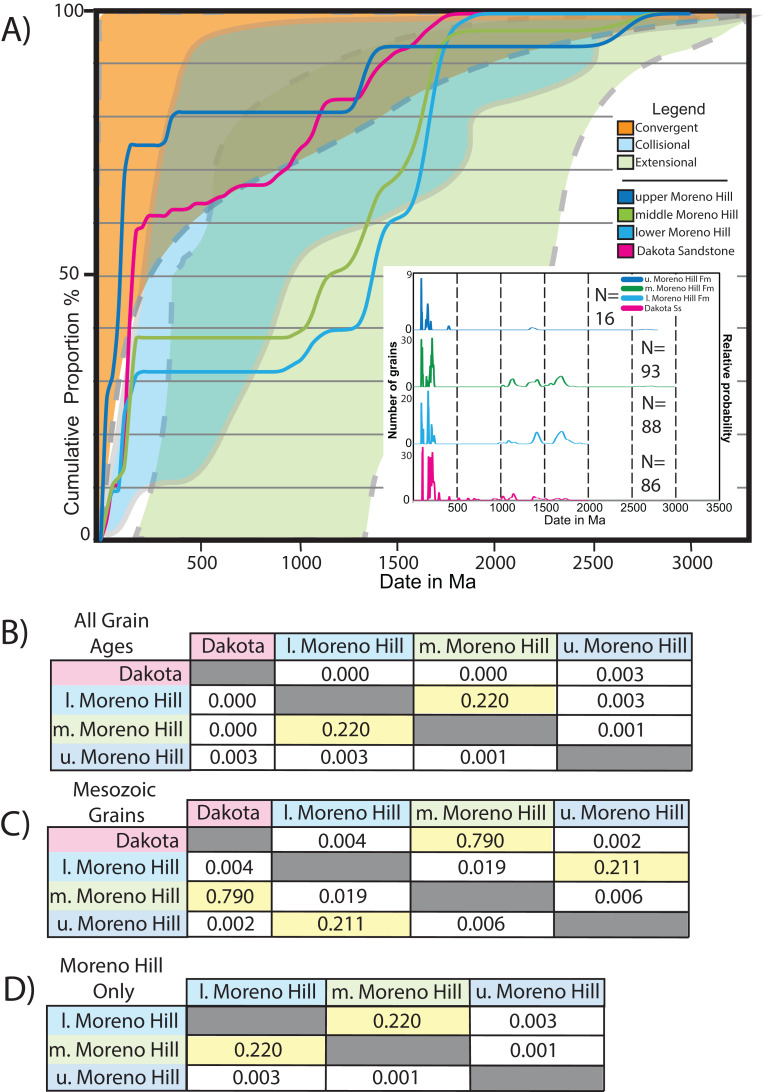
Continental setting via cumulative proportion curves. (A) Plot of the general field for Convergent, Collisional, and Extensional Basins based on and modified from Cawood, Hawkesworth & Dhuime (2012; Fig. 3, p. 877). (B–D) Plots display genetic relationships between samples; similarity is noted where *p*-values are >0.05 as shown graphically above. K–S analysis after Barbeau et al. (2009b).

The Kolmogorov–Smirnov test (K–S test) was applied to determine the likelihood that the age profiles of sampled zircons obtained from the Dakota Sandstone and from the lower, middle and upper members of the Moreno Hill are statistically similar (pass) or dissimilar (fail). In this study, we utilized a Cumulative Distribution Function (CDF) via date and its corresponding uncertainty with a 95 % confidence interval, in that *p*-values > 0.05 pass and < 0.05 fail the test (Fig. 7) (Dickinson & Gehrels, 2008; Barbeau et al., 2009a; Tucker et al., 2016, 2020).

When comparing all four samples based on a CDF across all reported dates spanning Precambrian-Proterozoic—Mesozoic-Cretaceous we note no statistical similarity between the underlying Dakota Sandstone and the overlying Moreno Hill (*p*-value = 0.00). Furthermore, with the same parameters, the lower and middle Moreno Hill samples have genetic similarity (*p*-value = 0.222), yet neither showed similarity with the upper Moreno Hill sample (*p*-values = 0.003 and 0.001). The lack of genetic similarity between Moreno Hill samples reflects (1) the limited number of recovered zircon grains from the upper Moreno Hill; (2) statistical effects of the 5% cutoff, and; (3) the very youthful multi-grain population between 89 and 88 Ma that is not present in the lower to middle Moreno Hill. This pattern is somewhat different when the K–S test is applied only to Mesozoic populations. For instance, the Dakota and the middle Moreno Hill have genetic similarity (*p*-value = 0.790), yet the Dakota fails to be significantly different from all other comparisons. When we compare only Moreno Hill samples, the lower and middle samples present genetic similarity (*p*-value = 0.220), and no similarity to the upper Moreno Hill (*p*-value = 0.003/0.001) (Fig. 7). Such subtle variances in confidence are potentially linked to variably geographically influenced drainage systems and/or to temporally dissimilar volcanic inliers within the westerly lying arc rather than to a single, enduring source (Fitz-Diaz, Hudleston & Tolson, 2011 and references therein).

The above described multi-faceted source terrane narrative is fairly complex; therefore, we sought to confirm these observations by utilizing the methods described by Cawood, Hawkesworth & Dhuime (2012), which links cumulative proportion curves with tectonic sources (Fig. 7). When results for dates between 0 and 3.5 Ga are plotted on cumulative proportion curves, all four samples variably plot between zones A (convergent), B (collisional), and C (extensional) confirming the complex detrital source terrane history (Cawood, Hawkesworth & Dhuime, 2012). By and large, all youthful populations (*n* < 100 Ma) from all four samples indicate a convergent margin (westerly lying arc); yet, weak genetic similarity between youthful samples indicates that these are likely derived from different volcanic inliers (Dakota and the middle Moreno Hill *p*-value = 0.790 and the lower and upper Moreno Hill *p*-value = 0.211, with all other comparisons failing the 0.05 significance threshold). It should be noted that with the 5% filter, only 16 grains were approved for the upper Moreno Hill, and should be treated as a proxy only. However, when all the samples are presented on a detrital zircon age probability plot, irrespective of the final grain count, all samples present a Foreland Basin spectrum (in agreement with Cawood, Hawkesworth & Dhuime, 2012, Figs. 1C and 1D, p. 876).

## Discussion

**Figure 8:**
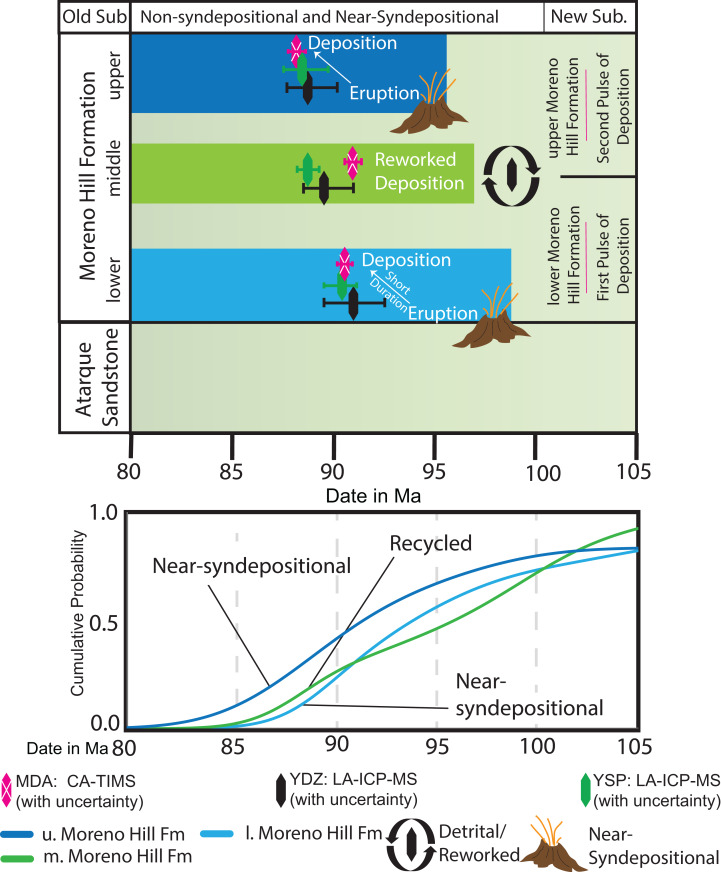
Sedimentation and zircon input. Plot displays temporal relationship between crystallization age and the delayed depositional input. Due to the similarity of the lower and middle Moreno Hill, this study finds evidence for a singular, longer-lived pulse of sediment input. Thereafter, a second phase with more youthful zircons, and a varied zircon history thus indicates a potential new subdivision of the Moreno Hill Formation into lower and upper members only. K–S analysis after Barbeau et al. (2009b).

This study sought to (1) refine the depositional age and duration of sedimentation of the Moreno Hill Formation; (2) provide newly calibrated linkages based on the revised temporal framework; and (3) determine likely source terranes. Our results demonstrate that emplacement of sediment into the Moreno Hill depo-center was diachronous, occurring in two distinct phases. The first phase of sedimentation occurred after 90.9 Ma and terminated before 88.6 Ma (lower to middle Moreno Hill), spanning the late Turonian to very early Coniacian (Fig. 8). The second phase of sedimentation occurred shortly thereafter 88.6 Ma (upper Moreno Hill), very early Coniacian (Fig. 8) (Cohen et al., 2013:v2020/03). This two-phased deposition is reflected in the results of the K–S test, with only the lower and middle Moreno Hill having genetic similarity. Therefore, a comparison of stratigraphic position and the resulting MDAs would strongly indicate that there was no synchronicity between sedimentation and the emplacement of detrital zircons in the middle Moreno Hill, rather that only the lower and upper Moreno Hill could potentially be nearer to syndepositional (Rossignol et al., 2019; Tucker et al., 2020). In light of the seemingly strong temporal relationship between the lower and middle members of the Moreno Hill, the current informal subdivision of the formation into its three members will be reassessed in a forthcoming manuscript.

**Figure 9:**
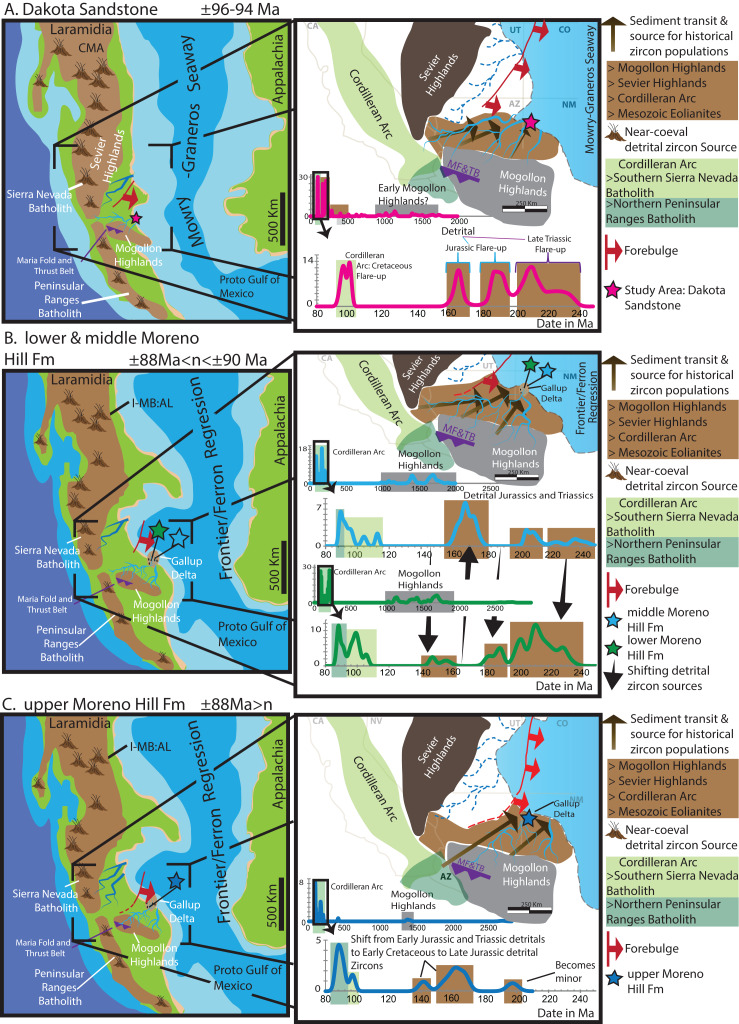
Reconstruction of the Moreno Hill depo-center. (A) Emplacement of the alluvial Dakota Sandstone after 96 Ma during the Mowry–Graneros Seaway with sediment transported via northeasterly draining fluvial systems. (B) Emplacement of sediment in the lower and middle Moreno Hill Formation, with youthful detrital zircons from the Cordilleran Arc accompanied by sediment and historical zircon populations from the Mogollon Highlands, along with surrounding (reworked) sedimentary blanket southeast of the Sevier Highlands and north of the Mogollon Highlands into the Gallup Delta. (C) Emplacement of sediment into the upper Moreno Hill depo-center with similar source terranes to the underlying units; yet, with the eastward migrating forebulge it is less likely the Sevier and its eastwardly adjacent sedimentary blanket to be a prominent source (now within the north-trending foredeep). Geophysical/paleogeographic maps are based on a synthesis of sources, namely Blakey (2014), Pecha et al. (2018), Lin, Bhattacharya & Stockford (2019), Miall & Catuneanu (2019), Tucker et al. (2020) and references therein.

This study notes that based on the depositional nature of detrital zircon (Gehrels, 2014), sedimentation into the Moreno Hill could have been entirely within the very early Coniacian; however, key pieces of evidence seemingly corroborate staggered pulses of detrital-rich sedimentation into the Moreno Hill depo-center. The first phase of sedimentation is well-documented to have co-occurred with anoxic paleosols and distinct coal horizons within a distal alluvial plain (Fig. 9) (McLellan et al., 1983a; Landis et al., 1985; Mack, 1992; Hoffman, 1996; Sweeney et al., 2009). On the other hand, the upper Moreno Hill is distinctly different, preserving paleosols generally indicative of subaerial conditions and aridification with co-occurring fluvial channel belts (Roybal, 1982; Campbell, 1984; Hoffman, 1996). While the complete lithological review of the Moreno Hill is the focus of a forthcoming manuscript, we wish to highlight the above-mentioned differences between the lower and upper Moreno Hill are supported by our own observations. The lower Moreno Hill is characterized by thickly interbedded dark fissile to blocky plant hash-rich mudrocks with associated coal seams, finely laminated siltstones, fine to medium-grained laterally discontinuous sandstones, and fine to coarsely-grained sublitharenitic to subarkosic laterally continuous multi-story planar and distinctly trough-cross-bedded ledge-forming sandstones more prevalent towards the upper parts of the member; whereas, the upper Moreno Hill is characterized by lighter-colored more fissile mudrocks, siltstones and lesser-occurring very finely to medium-grained subarkosic lenticular trough-cross-bedded sandstones (Campbell, 1984; Cook & Arkell, 1987) (details to be updated in forthcoming manuscript). Beyond local sedimentological differences, regional linkages can be utilized to corroborate this study’s interpretation of diachronous sedimentation. Based on regional biostratigraphic linkages of *C. woollgari woollgari* and either *M. labiatus* (Wolfe & Kirkland (1998, p. 304) or *M. mytiloides* (Kirkland, Smith & Wolfe (2005, p. 89)), the underlying diachronous Atarque Sandstone was emplaced during the latest early-Turonian to early-middle Turonian (~93.4 and ~92.5 Ma) (Kauffman et al., 1993; Cobban et al., 2006). Biostratigraphically controlled radiometric ^40^Ar/^39^Ar (sanidine) dates correlative with the lower Moreno Hill Formation are available from other diachronous units including the Ferron Sandstone, Utah, and from the Juana Lopez Member of the Mancos Shale in San Juan County, New Mexico (Obradovich, 1993; Cobban et al., 2006). Both dates have been re-calibrated to current standards, dating the bentonite beds within the *P. hyatti* and *P. macombi* Zones to 91.1 ± 0.5 Ma and 90.8 Ma ± 0.7, respectively (Fowler, 2017). A bentonite from within the *S. preventricosus* Ammonite Zone (Lower Coniacian), Marias River Shale in Montana, was dated and recalibrated to 88.9 ± 0.6 Ma, thus constraining the age of *Flemingostrea elegans* within the Mulatto Tongue of the Mancos Shale (which overlies the upper member-correlative Dilco Coal Member of the Crevasse Canyon Formation) to early Coniacian (Obradovich, 1993; Molenaar et al., 2002; Hook, 2010; Fowler, 2017).

Our MDA for the lower Moreno Hill corroborates this placement (within ±1 Ma) with the initial sedimentation into Moreno Hill depo-center occurring after 90.9 Ma (in agreement with Wolfe (1989), Obradovich (1993), Dyman et al., 1994, Wolfe & Kirkland (1998), Cobban et al. (2006), Molenaar et al. (2002), Meyers et al. (2012)). Furthermore, the emplacement of sediment into the upper Moreno Hill depo-center is slightly younger than the original estimates by Wolfe & Kirkland (1998) of the upper Turonian. This study interprets that sedimentation occurred after 88.6 Ma, the very early Coniacian (Cohen et al., 2013:v2020/03), in agreement with the supposition of Chin et al. (2019). Accordingly, based on our MDA, the basal upper Moreno Hill could yet be linked to the *S. preventricosus* (89.8 Ma) or *S. ventricosus* (88.8 Ma) Ammonite Zones (Elder & Kirkland, 1993; Ogg & Hinnov, 2012; Fowler, 2017). Therefore, the sedimentation processes of the upper Moreno Hill may have had more direct linkages with the Gallup Sandstone and Crevasse Canyon Formation than previously thought (McLellan et al., 1983a; Molenaar et al., 2002).

In light of the above interpretations and if it assumed the most recent revision of the Gallup Delta by Lin & Bhattacharya (2019) and Lin, Bhattacharya & Stockford (2019) is accurate, the Moreno Hill depo-center was likely a sedimentary conduit for the Gallup Delta. Seminal work by Lin, Bhattacharya & Stockford (2019) indicates that initial sedimentation in the Gallup System occurred near ±89.6 Ma for the Lower Gallup and terminated by ±88.4 Ma for the Upper Gallup (Lin, Bhattacharya & Stockford, 2019, Fig. 20), and geographically is placed at or just north to northeast of the Moreno Hill depo-center (Lin, Bhattacharya & Stockford, 2019, Figs. 2A and 2B). Therefore, based on this study’s initial findings, we interpret the lower Moreno Hill would have formed the proximal portions of the delta plain with co-occurring channel complexes, vast back swamps, and accumulations of water-saturated floodplain fines (Fig. 9). By approximately 88.6 Ma (but no older) the delta prograded further north by northeast Lin, Bhattacharya & Stockford, 2019, Fig. 20, p. 571, Sequence 5, 4, 3), which is reflected in the Moreno Hill sedimentation. The once delta plain shifted to a distal floodplain with the continued development of the fluvial complex and adjacent floodplain fines preserved in slightly more arid climatic conditions (Roybal, 1982; Campbell, 1984; Hoffman, 1996; this study). In a broader context, with the newly interpreted MDAs for the Moreno Hill, emplacement of sediment into the depo-center would have initiated during the latest phase of the Greenhorn continuing through the Frontier-Ferron regression (Mancos Seaway) (Kauffman, 1984, Blakey, 2014; Lowery et al., 2018, Fig. 4, p. 14; Miall & Catuneanu, 2019; and references therein). The revision of lithostratigraphic, sequence stratigraphic, and biostratigraphic ties are the focus of a forthcoming manuscript and is beyond the scope of this particular study. However, these findings thus far are in agreement with the regional framework(s) (Fig. 9) by Roberts & Kirschbaum (1995, Fig. 10, p. 23 and Fig. 13, p. 29) Pecha et al. (2018) and Lin, Bhattacharya & Stockford (2019).

The oldest detrital zircons in the Moreno Hill Formation are Precambrian and are interpreted as being from the uplifted and eroding Yavapai and Mazatzal blocks in the adjacent Mogollon Highlands. All Moreno Hill Formation samples are void of Paleozoic multi-grain populations. Triassic to middle Jurassic recycled grains and populations are interpreted as being from the Appalachian and Amarillo-Wichita uplifts (300–200 Ma) with younger grains being from westerly lying terranes within the early phases of the Cordilleran Arc and heavily-reworked multi-generational recycling of sedimentary blankets (aeolianites) lying east of the Sevier Highlands and north of the Mogollon Highlands (Dickinson & Gehrels, 2003, 2009a, 2009b; Lawton, Pollock & Robinson, 2003; DeCelles, 2004; Laskowski, DeCelles & Gehrels, 2013; Gehrels & Pecha, 2014). Late Jurassic—“mid-Cretaceous” zircon populations can be linked to igneous terranes in the southwestern portion of the Cordilleran Arc, specifically the western zones of the Sierra Nevada and Peninsular Ranges Batholiths (Laskowski, DeCelles & Gehrels, 2013; Tucker et al., 2020). Based on regional paleo-drainage reconstructions from southwest to northeast, we conclude that the most youthful (near-syndepositional) populations are most likely derived from the southeastern zone of the Sierra Nevada Batholith and the northeastern zone of the Peninsular Ranges Batholith (Roberts & Kirschbaum, 1995; Hildebrand & Whalen, 2014; Yonkee & Weil, 2015; Pecha et al., 2018; Lin, Bhattacharya & Stockford, 2019). If the Moreno Hill is compared to the Dakota Sandstone, our study confidently identifies a long-term shift in probable source terranes and is linked to an evolving catchment system. Specifically, the impacts of the eastward migration of the forebulge to the north (Currie, 1997; DeCelles, 2004; White, Furlong & Arthur, 2002; DeCelles & Coogan, 2006; Yonkee & Weil, 2015; Miall & Catuneanu, 2019) may have extended further south to southwest than previously recognized, thus creating a topographic high, which diverted drainage into the north (foredeep) or northeast (backbulge) (Figs. 9B and 9C). We interpret that as the forebulge migrated eastward, it slowly cut off westerly lying sources, including the Sevier Highlands and northern Sierra Nevada Batholith (Fig. 9). Volcaniclastic to volcanilithic-rich sediment that blanketed the Mogollon Highlands during eruption phases would have been eroded and mixed with other Mogollon Highland sediments and transported northeast to the Moreno Hill depo-center and the Gallup Delta. Temporally, the tectonic driver for sediment and resulting influence on drainage (southwest to northeast) can be confidently linked to the 90–86 Ma development of the Maria Fold and Thrust Belt (Spencer & Reynolds, 1990; Knapp & Heizler, 1990, Barth et al., 2004; Salem, 2009; Szwarc et al., 2015).

The Moreno Hill Formation preserves a globally unique, terrestrial vertebrate fauna that has been used alongside other key formations (e.g., the Cedar Mountain Formation (Cifelli et al., 1997; Kirkland et al., 1997; Zanno & Makovicky, 2013; Zanno et al., 2019), and the Cloverly Formation (Zanno & Makovicky, 2011; Farke et al., 2014)) to detangle the pace of ecological restructuring in western North America during the mid-Cretaceous (Nesbitt et al., 2019).

Specifically, the Moreno Hill Assemblage (sensu Nesbitt et al., 2019), which currently derives solely from the lower Moreno Hill Formation (Wolfe & Kirkland, 1998), has been used to pinpoint first and last appearance dates for a variety of key taxa including therizinosauroids, hadrosauroids, and ceratopsians (Wolfe & Kirkland, 1998; Kirkland & Wolfe, 2001; McDonald, Wolfe & Kirkland, 2010; Gates et al., 2011), and fills in biodiversity data otherwise only supplemented temporally by the more poorly categorized Straight Cliffs Formation regionally (Titus, Roberts & Albright, 2013; Albright & Titus, 2016). Although Wolfe & Kirkland (1998) suggest a middle—upper Turonian age for the lower Moreno Hill Formation based on ammonite biostratigraphy (Molenaar et al., 2002), recent paleontological studies have used an early middle Turonian age (~92 Ma) in taxon descriptions (Nesbitt et al., 2019). Our MDA of 90.9 Ma from the Moreno Hill Assemblage compares well with the temporal framework of Wolfe & Kirkland (1998) and suggests that taxon ages should be refined to be approximately 1 million years younger than previously recognized, whereas, the limited fossils recovered from the upper Moreno Hill are Coniacian.

## Conclusion

This study presents a newly calibrated chronostratigraphic framework for the Moreno Hill Formation exposed within the Zuni Basin, New Mexico. By coupling CA-TIMS and LA-ICP-MS data, we identify that emplacement of the most reliable youthful zircon populations preserved within the Moreno Hill depo-center occurred in two distinct phases. The first pulse of deposition occurred after 90.9 Ma (lower Moreno Hill), and the second pulse of sediment emplacement occurred after 88.6 Ma. Based on the principle of detrital zircon, the emplacement of the Moreno Hill is diachronous, Turonian/Coniacian. Based on LA-ICP-MS data this study was able to detangle a complex history of detrital input and confidently identify likely volcanic source terranes. Youthful populations are interpreted to derive from the westerly Cordilleran Arc (Phase C), and more likely Peninsular Ranges Batholith and the southernmost to central Sierra Nevada Batholith (Fig. 9). Reworked volcanic detritus and co-occurring detrital sediment from the Maria Fold and Thrust Belt and the Mogollon Highlands were enriched with Cordilleran Arc detritus and transported via fluvial complexes to the developing Gallup Delta (Fig. 9). The Moreno Hill is interpreted to be the proximal delta plain and distal fluvial system to the Gallup Delta (Hutsky & Fielding, 2016; Lin & Bhattacharya, 2019; Lin, Bhattacharya & Stockford, 2019). Our future investigations into the Moreno Hill Formation will seek to couple this newly calibrated temporal framework with that of historically significant lithostratigraphic and biostratigraphic records, which are now somewhat juxtaposed with our current results. Future work will also seek to provide a comprehensive review of the Moreno Hill sedimentary system including a stratigraphic revision of its current subdivision. Finally, these efforts present a novel temporal framework for the Moreno Hill assemblage that will allow refined comparisons with early-Late Cretaceous terrestrial ecosystems of the Western Interior Basin.

## Supplemental Information

10.7717/peerj.10948/supp-1 Supplemental Information 1 U/Pb LA-ICPMS isotopic data. Results for all detrital zircon samples, including isotope and concentration data. Th (in ppm) is Thorium concentration measured in parts per million; U (in ppm) is Uranium concentration in parts per million. Both 207Pb/235U and error calculated at ±2σ and 206Pb/238U and error calculated at ±2σ were used to calculate the error correction (Error Corr.). The ratio values for 206Pb/238U, 207Pb/206Pb, and 207Pb/206Pb with all error calculated at ±2σ. Omitted grains that failed the 5% filter based on a 207Pb/235U and 206Pb/238U are included within the final tables, but are indicated in red text.

## Additional Information and Declarations

### Field Study Permissions

The following information was supplied relating to field study approvals (i.e., approving body and any reference numbers): Land access was provided by the Bureau of Land Management (New Mexico); permit number "Moreno Hill Survey NM17-02S".

### Data Availability

The following information was supplied regarding data availability: Both CA-TIMS and LA-ICPMS U-Pb detrital zircon isotope data are available in the Supplemental Files.
